# A Dangerous Remedy

**Published:** 1883-04

**Authors:** 


### A DANGEROUS REMEDY.

Here is a formula which has been frequently prescribed for toothache:

Spts. Chloroformi,

" Aeth. Sulph.,

" Camph.,

Tr. Opii.,

" Iodinii, - - - *aa* f. ℨ i.

That is a nice compound to place in the hands of an ignorant person, with no other directions than to wet a pledget of cotton with the mixture and place in the aching tooth. It ought to be efficacious, certainly, if there is any virtue in drugs, and if applied by an intelligent practitioner it might be safe; but to place such a prescription in the hands of careless or uninstructed people is little short of criminal recklessness.

